# Empty spiracles homeobox genes EMX1 and EMX2 regulate WNT pathway activation in sarcomagenesis

**DOI:** 10.1186/s13046-021-02048-9

**Published:** 2021-08-07

**Authors:** Manuel Pedro Jimenez-García, Antonio Lucena-Cacace, Daniel Otero-Albiol, Amancio Carnero

**Affiliations:** 1 Instituto de Biomedicina de Sevilla (IBIS), Hospital Universitario Virgen del Rocío, Universidad de Sevilla, Consejo Superior de Investigaciones Científicas, Sevilla, Spain; 2 CIBER de Cancer, IS Carlos III, Madrid, Spain; 3 Present address: Department of Cell Growth and Differentiation, Center for iPS Cell Research and Application, Kyoto University, Kyoto, Japan; 4 Instituto de Biomedicina de Sevilla/HUVR/CSIC, Hospital Universitario Virgen del Rocío, Avda. Manuel Siurot s/n, 41013 Sevilla, Spain

**Keywords:** Wnt pathway, b-catenin, EMX, Sarcoma, Cancer, Cancer stem cells

## Abstract

**Background:**

Sarcomas are a very heterogeneous group of tumors with intrinsic developmental programs derived from the cell of origin. This implies a functional hierarchy inside tumors governed by sarcoma stem cells. Therefore, genetic and/or epigenetic changes profoundly affect the biology of sarcoma tumor stem cells. *EMX* genes are proposed to be transcription factors that are involved in the sarcomagenesis process, regardless of the neural or mesodermal embryological sarcoma origin. It has been shown that *EMX1* or *EMX2* overexpression reduces tumorigenic properties, while reducing the levels of these genes enhances these properties. Furthermore, it has been shown that *EMX* genes decrease the expression of stem cell regulatory genes and the stem cell phenotype. Taken together, these results indicate that the *EMX1* and *EMX2* genes negatively regulate these tumor-remodeling populations or sarcoma stem cells, acting as tumor suppressors in sarcoma.

**Methods:**

Bioinformatic analysis, quantitative mRNA and protein expression analysis, cell models of sarcoma by ectopic expression of EMX genes. By cell biology methods we measured tumorigenesis and populations enriched on stem cell phenotypes, either in vitro or in vivo.

**Results:**

In this work, we showed that the canonical Wnt pathway is one of the mechanisms that explains the relationships of *EMX1*/*EMX2* and stem cell genes in sarcoma. The Wnt-*EMX1*/*EMX2* relationship was validated in silico with sarcoma patient datasets, in vitro in primary derived sarcoma cell lines, and in vivo. EMX expression was found to negatively regulate the Wnt pathway. In addition, the constitutive activation of the Wnt pathway revers to a more aggressive phenotype with stem cell properties, and stemness gene transcription increased even in the presence of *EMX1* and/or *EMX2* overexpression, establishing the relationship among the Wnt pathway, stem cell genes and the *EMX* transcription factors.

**Conclusions:**

Our data showed that Empty Spiracles Homeobox Genes EMX1 and EMX2 represses WNT signalling and activation of WNT pathway bypass EMX-dependent stemness repression and induces sarcomagenesis. These results also suggest the relevance of the Wnt/b-catenin/stemness axis as a therapeutic target in sarcoma.

**Supplementary Information:**

The online version contains supplementary material available at 10.1186/s13046-021-02048-9.

## Background

Sarcomas are a very heterogeneous group of tumors including more than 40 different histopathological subtypes, giving complexity to their study. They affect a low percentage of the population and often appear as benign tumors; however, their malignant form is highly aggressive. Furthermore, surgical resection (with the addition of radiotherapy in some cases) of the primary tumor is still the only curative modality, and only few chemotherapeutic options can induce complete remission in most sarcoma subtypes [1, 2].

Different sarcomas are proposed to develop from different cells of origin, including mesenchymal stem cells, which are naturally heterogeneous; mesenchymal committed progenitors; neural crest-derived stem cells; and muscle satellite cells, as in rhabdomyosarcoma [3, 4] The different origins impinge on the heterogeneity of the different types of sarcoma, and genetic and epigenetic changes associated with sarcomagenesis further impact the biology of sarcoma stem cells and derived tumors. Therefore, sarcomagenesis and the underlying biology of sarcoma stem cells define the later heterogeneity of these tumors. However, some mechanisms of stemness maintenance are similar among diverse sarcoma types, such as Sox2 activation and deregulation of the canonical Wnt pathway [3]. In this regard, many sarcomas originate from the differentiation of neural crest-derived pluripotent cells [5, 6], and certain genes involved in the differentiation of this main embryological feature, such as Empty Spiracles Homeobox EMX1/EMX2 tumor suppressor genes, have previously been related to repression of sarcomagenesis [4], especially maintaining stem cells in a quiescent state [7–10].

The EMX homeodomain proteins are members of the EMX family of transcription factors and have been related to not only sarcoma but also melanoma [11], in which the originating melanocytes are also derived from the neural crest, as well as solid tumors of epithelial origin and tumors of mesodermal origin, such as glioblastoma [7, 12–19]. In the tumors tested to date, the restoration of EMX1/2 expression levels suppresses cell proliferation and the invasive phenotype; it also sensitizes lung cancer cells to treatment, suggesting that EMX1/2 may be tumor suppressor genes [15–17]. Therefore, these EMX proteins have been reported to be involved in the regulation of various biological processes, such as cell proliferation, migration, and differentiation during the development of the brain and neural crest [7–9]. However, many sarcoma cells and tumors still maintain high levels of Emx1/Emx2 protein expression [4], suggesting the existence of other active pathways that bypass the tumor suppressive roles of EMX1/EMX2 in the initiation and progression of human sarcoma.

We previously reported that downregulating EMX protein expression in mouse KO models favors sarcomagenesis. Therefore, the bypass of EMX proteins, or a hierarchy that imposes activity that overcomes EMX stemness-derived repression, might contribute to sarcomagenesis (or tumorigenesis in general) in an essential manner.

Although little is known about the downstream transcriptional targets of EMX proteins, it has been suggested that the isoform EMX2 is a direct inhibitor of Wnt-1 expression via an EMX2 DNA-binding site found in a Wnt-1 gene enhancer. The MiTF isoform present in melanocytes (MiTF-M) is known to be positively regulated by the Wnt pathway, and both Wnt1 and Wnt3a are known to be important in determining the fate of neural crest-derived cells, including melanocytes [20–22]. Additionally, a negative correlation with the Wnt pathway was preliminarily established in lung and gastric cancers.

The WNT signaling pathway regulates a wide range of important physiological processes, such as cell polarity, cell fate, apoptosis, proliferation and dedifferentiation of CSCs [23–26]. WNT deregulation is associated with many diseases, including cancer [27, 28]. The WNT pathway can be subdivided into canonical and noncanonical pathways. The canonical pathway leads to derepression of β-catenin through the LZD-LRP5/6 regulator complex. Noncanonical signaling leads to the activation of the PCP, RTK and Ca + 2 signaling cascades [29, 30]. In canonical WNT signaling, in the absence of Wnt ligands, b-catenin is phosphorylated and degraded. In the presence of Wnt ligands, activation of the WNT pathway occurs, and b-catenin is not degraded but rather released from the Axin complex to translocate into the nucleus, where it enhances the recruitment of coactivators to activate transcription [24–26, 28]. Aberrant activation of the WNT/b-catenin pathway by different alterations is found in many epithelial cancers [31–36] and sarcomas [37–41]. Therefore, it has been proposed that targeting the WNT pathway in sarcomas might be a suitable strategy, since cells from sarcoma tumors might maintain a deregulated WNT pathway.

The role of the Wnt pathway in sarcomas derived from the mesoderm or neural crest has not yet been elucidated. Nor have the molecular pathways altered by Wnt that are dependent on EMX1/EMX2 levels been studied in depth. In the present work, we show that EMX homeodomain proteins negatively regulate the Wnt pathway and that independent activation of the Wnt pathway bypasses this EMX-induced arrest and can initiate sarcomagenesis.

## Methods

### Ethics approval

All methods were performed in accordance with the relevant guidelines and regulations of the Institute for Biomedical Research of Seville (IBIS) and University Hospital Virgen del Rocio (HUVR). Animal experiments were performed according to the experimental protocol approved by HUVR Animal Ethics Committee (CEI 0309-N-15).

### Availability of supporting data

No datasets were generated during the current study. The datasets analyzed during the current study are publicly available in the different repositories. See Sarcoma databases used in this study section.

### Sarcoma databases used in this study

The following sarcoma databases, see their accession codes or manuscript of firs citation, were used to perform analyzes of EMX1/EMX2 expression levels and genes related to stem cell properties in the public databases of human embryonic cells (hESC) and human embryonic cells. Human Induced Pluripotent Stem Cells (iPSC):

- Yamanaka, GEO ID: GSE9561; Narita, M., et al. Cell 131, 861–872 (2007).
- Thomson, GEO ID: GSE15148; Junying, Y et al., Science. 324, 797–801 (2009).

The following sarcoma databases, with their accession codes and/or published papers where they were first described, were used to perform the correlation analyzes and additionally transcriptomic analyzes:

1. General sarcoma databases:
  TGCA (*N* = 259), Soloway, M. G., et al. Cell 171, 950–965.e28 (2017).
  Boshoff (*N* = 96), Henderson, S. R., et al. Genome Biol. 6, R76 (2005).
  Filion (*N* = 137), Filion, C., et al J. Pathol. 217, 83–93 (2009).
2. Ewing sarcoma databases:
  Delattre (*N* = 117), GEO ID: GSE34620
  Savola (*N* = 117), GEO ID: GSE17679
3. Osteosarcoma databases:
  Kuijjer (*N* = 127), GEO ID: GSE30385
4. Rhabdomyosarcoma databases:
  Davicioni (*N* = 147), Davicioni, E., et al. Cancer Res. 66, 6936–6946 (2006).
  Barr (*N* = 58), GEO ID: GSE66533
5. Rhabdoid sarcoma databases:
  Foreman (*N* = 71), GEO ID: GSE35493
  Perlman (*N* = 53), GEO ID: GSE11482
6. Head and neck tumor databases:
  TGCA (*N* = 520), Lawrence, M. S., et al. Nature 517, 576–582 (2015).

The analysis of the expression levels of EMX1/EMX2 was carried out with the indicated databases, since they are comparable between them because they were generated with the same platform and normalized with the same method. The expression values of R2-Genomics publicly available data platform were exported to the GraphPad-PRISM 6 program, with which the statistical study was carried out with the Student’s t-test.

To identify the genes whose expression correlated positively or negatively with the expression of EMX1/EMX2, the correlation between the EMX genes and the genes belonging to the KEGG (Kyoto Encyclopedia of Genes and Genomes) was analyzed: “Wnt signaling pathway”, “Notch pathway”, “Hippo pathway”, “microRNAs in cancer”, “cancer signaling pathways” and “signaling pathways that regulate stem cell pluripotency”, present in the R2 platform. These correlations were examined in the indicated databases and only those genes that correlated with EMX1/EMX2 were selected with a *p*-value less than 0.05 and Pearson’s correlation coefficient (R) equal to or greater than + 0.3 in positive correlations, and less than -0.3 in negative correlations.

Venn diagrams were represented with the online tool of the Bioinformatics and Evolutionary Genomics group of the University of Ghent (Belgium) (http://bioinformatics.psb.ugent.be/webtools/Venn/). The gene lists obtained at the intersection of the Venn diagram were sorted by KEGG and ordered by *p*-value.

### Transfections and plasmids

Subconfluent cells were transfected with TransIT-X2 reagent (Mirus) according to the manufacturer’s instructions. At 48 h, the cells were seeded in 10-cm plates with medium containing the appropriate selection drug (100–450 μg/ml G418, 0.25–0.4 μg/ml puromycin or 0.25–0.4 μg/ml blasticidin). Cells were transfected with the plasmids indicated in Supplementary Table 1:

### Cell culture

The cell lines used were generated in the laboratory as previously reported [42–44] or obtained from the ECACC commercial repository. No further authentication was conducted by the authors. The cells were negative for mycoplasma. The cell lines were maintained in DMEM, F10 medium or RPMI medium (AQmedium; Sigma), as indicated, supplemented with 10% fetal bovine serum (FBS) (Gibco), penicillin, streptomycin and fungizone (Sigma).

### Tumorigenesis assays

Proliferation assays, clone formation assays, growth assays in soft agar and cell migration and invasion assays were performed as indicated previously [45, 46]. Briefly,

#### Proliferation assay

One thousand cells were seeded in 35 mm wells in triplicate. At 24 h (day 0) the cells of the first point were fixed and every 48 h. The medium of the plates that remained in culture was changed every 2 days. Cells were fixed with 0.5% glutaraldehyde and stained with 1% crystal violet. After the plates were washed and allowed to dry, the crystal violet was dissolved in 15% acetic acid and the relative number of cells was quantified by measuring the absorbance of the crystal violet at 595 nm. The values were represented referring to day 0.

#### Clonability assay

Cells were seeded at low density, 1000 cells in triplicate 10 cm plates and cultured under standard conditions. The medium was changed twice a week and after 15 days, they were fixed with 0.5% glutaraldehyde and stained with 1% crystal violet. After washing, the number and size of the colonies were quantified.

#### Growth on soft agar

To measure anchor independent growth, 100,000 cells were resuspended in 1.4% low melting point agarose (iNtRON Biotechnology) with culture medium, in triplicate. This medium with the cells was placed on top of a pre-solidified 2.8% agar medium base in 35 mm wells. After a 24 h incubation at 37 °C, 3 ml of medium was added to each well, which was changed twice a week. After one month, photos were taken with an inverted microscope (Olympus IX-71), stained with crystal violet 0.05% and the number of colonies was counted.

#### Invasion assay

2.25 × 104 cells/well were seeded in serum-free medium in a Boyden chamber-type cell invasion assay (Cell Biolabs). 500 µl of culture medium supplemented with 10% FBS and 2% FBS was added to the lower well of the invasion chamber. They were incubated at 37 °C in a 5% CO2 atmosphere. After 24 and 48 h after seeding, the bottom of each well of the chamber was swabbed to remove non-migrating cells. An inverted microscope (Olympus IX-71) was used to obtain the photographs. To measure the amount of cells that could migrate, we transferred each insert to a clean well containing 400 µl of a cell staining solution, solubilized with the extraction solution provided by the kit, and measured at 595 nm.

### Tumorsphere assay

A total of 1 × 10^3^ cells were seeded in triplicate in 24-well ultra-low-attachment plates (Costar) containing 1 ml of MammoCult basal medium (Stem Cell Technologies) supplemented with 10% MammoCult proliferation supplement, 4 μg/ml heparin, 0.48 μg/ml hydrocortisone, penicillin and streptomycin. After 5–10 days, depending on the cell line, the number of primary tumorspheres formed was measured using an inverted microscope (Olympus IX-71). Subsequently, secondary tumorspheres were generated by collecting the medium containing the primary tumorspheres, centrifuging for 5 min at 900 rpm (91 g), trypsinizing for 5 min, and centrifuging again at 900 rpm (91 g) for 5 min. Finally, the cells were resuspended in the same complete supplemented MammoCult medium, and 1 ml per well was seeded in a 24-well low-attachment plate. The plates were incubated for 4–5 days, and the number and size of the tumorspheres were again evaluated and quantified.

### Single-cell tumorsphere assay

Single cells were individually seeded through cell sorting with a FACSJazz flow cytometer (BD Biosciences) into 96-well ultra-low-attachment plates containing 1 ml of MammoCult basal medium (Stem Cell Technologies) supplemented with 10% MammoCult proliferation supplement, 4 μg/ml heparin, 0.48 μg/ml hydrocortisone, penicillin and streptomycin. After 30 days, the number of individual primary tumorspheres formed was measured using an inverted microscope (Olympus IX-71).

### Analysis and classification of clonal phenotypes

Cells were seeded at a low density, 100 or 1000 cells depending on the cell line, in triplicate in 10-cm plates and cultured under standard conditions. The medium was changed twice per week. After 10–15 days, once clones had formed, the morphology of each clone was observed under an inverted microscope (Olympus CKX41). Finally, each type of clone was counted based on the following classification: holoclone (more compact and rounded clone with a higher percentage of cancer stem cells), meroclone (clone with a more irregular cell arrangement at the edges and a lower proportion of cancer stem cells), and paraclone (greater separation of cells from each other due to a greater degree of differentiation and lower percentage of cancer stem cells) [47, 48].

### Marker analysis by flow cytometry

A previously trypsinized suspension of 10^6^ cells was resuspended in 125 µl of PBS containing 2% FBS and 5 mM EDTA. This suspension was then blocked by adding 12.5 µl of blocking agent (Miltenyi Biotec) and incubated for 10 min on ice. An anti-CD133 antibody conjugated to the fluorochrome phycoerythrin (PE) (MACS) was then added at a 1:25 dilution and incubated for 30 min on ice in the dark. A tube containing 1–106 cells was left unlabeled with the antibody to use as a negative control for staining. After the end of the incubation period, the cells were washed 2 times with PBS containing 2% FBS and 5 mM EDTA. Subsequently, the cells were centrifuged for 5 min at a speed of 1000 rpm (112 g) and resuspended in 500 µl of PBS containing 2% FBS and 5 mM EDTA. Finally, the cell suspension was examined on a Canto II analytical cytometer (BD Biosciences), with the cells separated into positive and negative populations based on staining for the CD133 marker in certain cases.

### Total RNA extraction

The total RNA of the cell lines, of the tumorspheres and of the pulverized xenografts, were purified with the miRNeasy kit (Qiagen) for 5•105—2•106 cells. To the monolayer cells washed with PBS, 1 ml of PBS was added and using a scraper the cell lysate was extracted, deposited in a 1.5 ml tube and the sample was centrifuged for 5 min at 112 g, discarding the supernatant. The tissue was homogenized with the QIAzol lysis solution (Qiagen) to lyse the cells or the tumorspheres. Then 140 µl of chloroform was added, the solution was vortexed and incubated for 5 min. Subsequently, it was centrifuged for 15 min at 4ºC and 16,128 g to separate the different phases. The supernatant was transferred to a new tube and 1.5 volumes of absolute ethanol were added to precipitate the RNA. Subsequently, the entire volume was transferred to a minicolumn and centrifuged for 30 s at 7,168 g. The liquid was discarded, the RNA wash solution was added to the column and centrifuged again for 30 s at 7,168 g. The tube was then emptied and DNase I treatment was performed for 15 min at 25 °C. After the wash solution was added to the column, it was centrifuged for 30 s at 7,168 g. The last wash of the column was performed with the RNA wash solution and centrifuged for 2 min at 16,128 g. The collection tube was removed by another and centrifuged 1 min at maximum revolution to remove any remaining wash solution. Finally, the RNA was eluted with nuclease-free water. Later it was quantified by optical density (Nanodrop).

### Reverse transcription and polymerase chain reaction (RT-PCR)

Reverse transcription was performed with the High-Capacity cDNA Reverse Transcription Kit (ThermoFisher Scientific). 2 µl of 10X reverse transcription buffer, 0.8 µl of 25X deoxyribonucleotide triphosphate (dNTPs), 2 µl of 10X random sequence primers, 1 µl of reverse transcriptase and 4.2 µl of free water were mixed on ice. This 10 µl total volume mixture was added to a PCR tube containing 10 µl with 3 µg of total RNA diluted in nuclease-free water to complete the reaction volume. The PCR tube was then placed in a thermal cycler to carry out the reverse transcription reaction. The reaction consisted of the following steps: 10 min at 25 °C, 120 min at 37 °C, and 5 min at 85 °C. Once the reaction was finished, the complementary DNA (cDNA) was stored at -20 °C until use.

### Quantitative real-time PCR (qPCR)

The detection of changes in gene expression was performed with cDNA from reverse reverse transcription. To carry out the qPCR, the following quantities were added per well in 384-well plates (ThermoFisher Scientific): 2 μL of cDNA (1 µg), 5 μL of mixture for qPCR GoTaq® 2X (Promega) and 0.5 µl of TaqMan 20X Assay (Applied biosystem), containing the primers and probe for the mRNA of interest. The different TaqMan probes used in this thesis are described in Supplementary Table 2. The qPCR was performed in the ABI Prism 7900HT thermal cycler (Applied Biosystems). The PCR program consisted of 10 min at 95 °C, 40 cycles of 15 s at 95 °C, and 1 min at 60 °C. The analysis of the relative changes in gene expression was performed with the threshold cycle comparative method (ΔΔCt) and with the RQ manager program (Applied biosystem). To normalize the samples, the GAPDH gene was used as endogenous control. Each reaction was done in triplicate and 7 independent experiments were performed for each gene tested. A negative control sample (no cDNA) was introduced into each experiment.

We used the probes as indicated in Supplementary Table 2.

### Protein isolation and Western blot analysis

Western blots were performed as previously described. Membranes were incubated with the following primary antibodies (Supplementary Table 3):

Proteins were detected using an ECL detection system (Amersham Biosciences) and a Bio-Rad Chemidoc Touch. The Image Lab 5.1 program (Bio-Rad) was used to quantify protein bands. Each band was selected individually, and values are reported relative to a loading control on the same membrane, which was usually α-tubulin.

### Statistical analysis

We used the GraphPad Prism 6 software for all statistical analyses of the experiments performed with cell lines and the in vivo experiments. To analyze the differences observed in all the functional or transcriptomic tests carried out with the cell lines models transfected with a cDNA sequence for EMX1, EMX2, CTNNB1, shRNAs or different control vectors, xenograft and tumoresphere models generated we used Student’s t-test for unpaired samples or Student’s t-test with Welch's correction for unequal population variances, but the assumption of normality is maintained. To determine statistical significance from survival graphs, we used a Chi-square test (log-rank test). P values less than 0.05 were considered statistically significant and are represented according to the following classification: *p* < 0.05 (*), *p* < 0.01 (**) and *p* < 0.001 (***).

### Student’s t-test

In order to address the gaussian distribution of results analyzed by Student’s t-test, Welch’s correction were performed to get raw data throughout GraphPad Prism 6 software. Student's t-test assumes that the two populations being compared are normally distributed with equal variances, on the other hand, Welch’s t-test is designed for unequal population variances, but the assumption of normality is maintained. With unpaired t tests, when comparing the means between two different independent groups, all columns of data used are assumed to be normal, and were be tested either individually or jointly if it is assumed equal variance and tested the residuals. So that, all the experimental conditions were compared two by two to get them all analyzed, always referring to the EV cell model in each experimental case. T tests are robust to non-normal data with large sample sizes, meaning that as long as enough data is collected, only substantial violations of normality need to be addressed. GraphPad Prism 6 software offers four normality test options: D'Agostino-Pearson, Anderson–Darling, Shapiro–Wilk and Kolmogorov–Smirnov. Each of the tests produces a p-value that sums up the results. If the p-value is not significant, the normality test was ‘passed’. While it’s true we can never say for certain that the data came from a normal distribution, there is evidence that the data may be normally distributed.

## Results

### Correlations of EMX1 and EMX2 expression with genes in the Wnt pathway and other signaling pathways regulating the stem cell phenotype

**Fig. 1:**
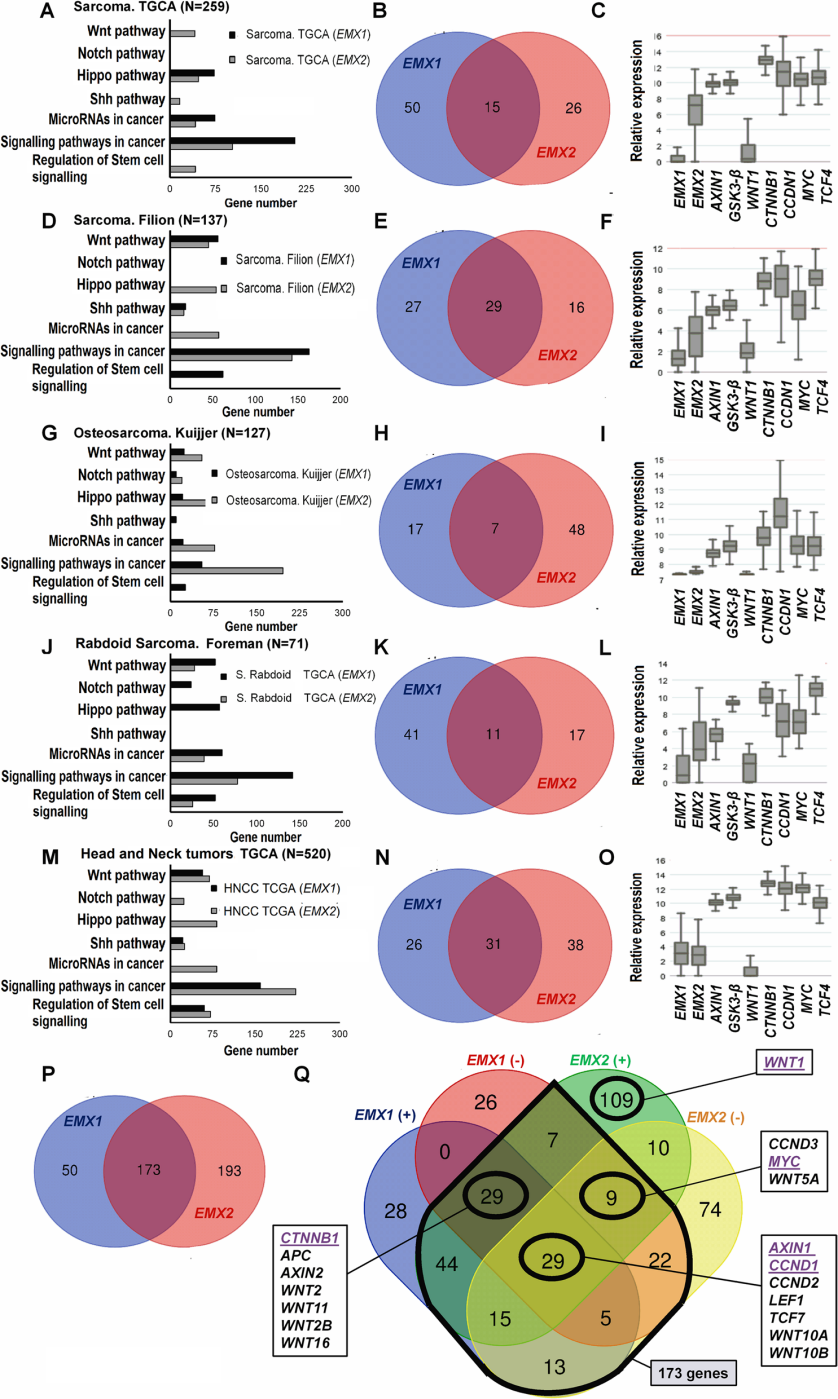
EMX1 and EMX2 expression correlated with effectors of the Wnt pathway in samples from sarcoma patients. **A**, **D**, **G**, **J**, and **M** Bar charts showing the classification of EMX1 and EMX2 target genes by KEGG analysis in sarcoma patient databases. The Y-axis represents the KEGG pathways related to the regulatory pathways of stem cells with a *p*-value < 0.05. **B**, **E**, **H**, **K** and **N**, Venn diagrams of the regulatory genes of the Wnt pathway correlated with EMX1 and EMX2. **P** and **Q** Venn diagrams encompassing all analyzed sarcoma datasets with respect to EMX1 and EMX2 correlations. **C**, **F**, **I**, **L** and **O** Relative expression of the positive prominent effectors WNT1, CTNNB1, MYC and CCND1 and the negative effectors AXIN1 and GSK3-β

The results obtained in our cell model showed a reduction in stem cell characteristics when EMX1 or EMX2 was overexpressed in primary sarcoma cell lines. To examine whether this effect was observed in sarcoma patients, we analyzed the correlations between the expression of EMX1/EMX2 and the expression of genes in signaling pathways regulating the phenotype of stem cells in thePerlman (*N* = 53), and head and neck tumors: TGCA (*N* = 520) datasets. In 11 sarcoma datasets available on the genomic analysis platform R2-Genomics (including general sarcoma: TGCA (*N* = 259), Boshoff (*N* = 96), Filion (*N* = 137); Ewing’s sarcoma: Delattre (*N* = 117), Savola (*N* = 117); osteosarcoma: Kuijjer (*N* = 127); rhabdomyosarcoma: Davicioni (*N* = 147), Barr (*N* = 58); rhabdoid sarcoma: Foreman addition), the antecedents related to the EMX genes and one of the regulatory pathways for the properties of stem cells were taken into account, namely, the canonical Wnt/β-catenin pathway. Specifically, Emx2 interacts in a neural context with a genomic region that enhances the expression of Wnt1, blocking the translation of Wnt1, a receptor in this pathway [49]. Additionally, in solid lung, gastric and melanoma tumors, inactivation of the canonical Wnt pathway has been suggested to occur in the context of EMX2 overexpression [11, 15–17]. To extrapolate these results to the context of sarcoma, a correlation analysis of the EMX1 and EMX2 genes with the Wnt pathway and the signaling pathways regulating the stem cell phenotype was performed (Fig. 1).

As a result, correlated genes in the regulatory pathways of the properties of stem cells were identified, which were classified according to the KEGG (Kyoto Encyclopedia of Genes and Genomes) results for each with a p-value less than 0.05. Specifically, the KEGG pathways of Wnt, Notch, Hippo and Shh were significant. Only datasets in which there were statistically significant correlations of both EMX1 and EMX2 with the Wnt pathway are represented (Fig. 1). A Venn diagram for Wnt pathway regulatory genes that were positively and negatively correlated with EMX1 and EMX2 with a p-value less than 0.05 and a Pearson's correlation coefficient (R) equal to or greater than the absolute value 0.3 was generated for each dataset (Fig. 1B, E, H, K and N). Additionally, a Venn diagram for all Wnt pathway regulatory genes that were positively and negatively correlated with EMX1 and EMX2 in all the analyzed datasets was generated (Fig. 1P, Q). From the intersection of the correlations of the EMX1 and EMX2 genes, 6 effectors were chosen from the 173 genes based on statistical significance. The 6 effectors of the Wnt pathway were divided into those that exert a positive or negative effect on the activation of the pathway. The positive effectors were WNT1, CTNNB1, MYC and CCND1, and the negative effectors were AXIN1 and GSK3-β. Additionally, the relative expression levels of the 6 positive and negative effectors of the Wnt pathway are indicated for each represented dataset (Fig. 1C, F, I, L and O). These results established activation of the Wnt pathway in a generalized context of inactivation of the EMX1 and EMX2 genes.

### Characterization of the activation state of the Wnt pathway in EMX1/EMX2-overexpressing and EMX1/EMX2-silenced sarcoma cell models

**Fig. 2:**
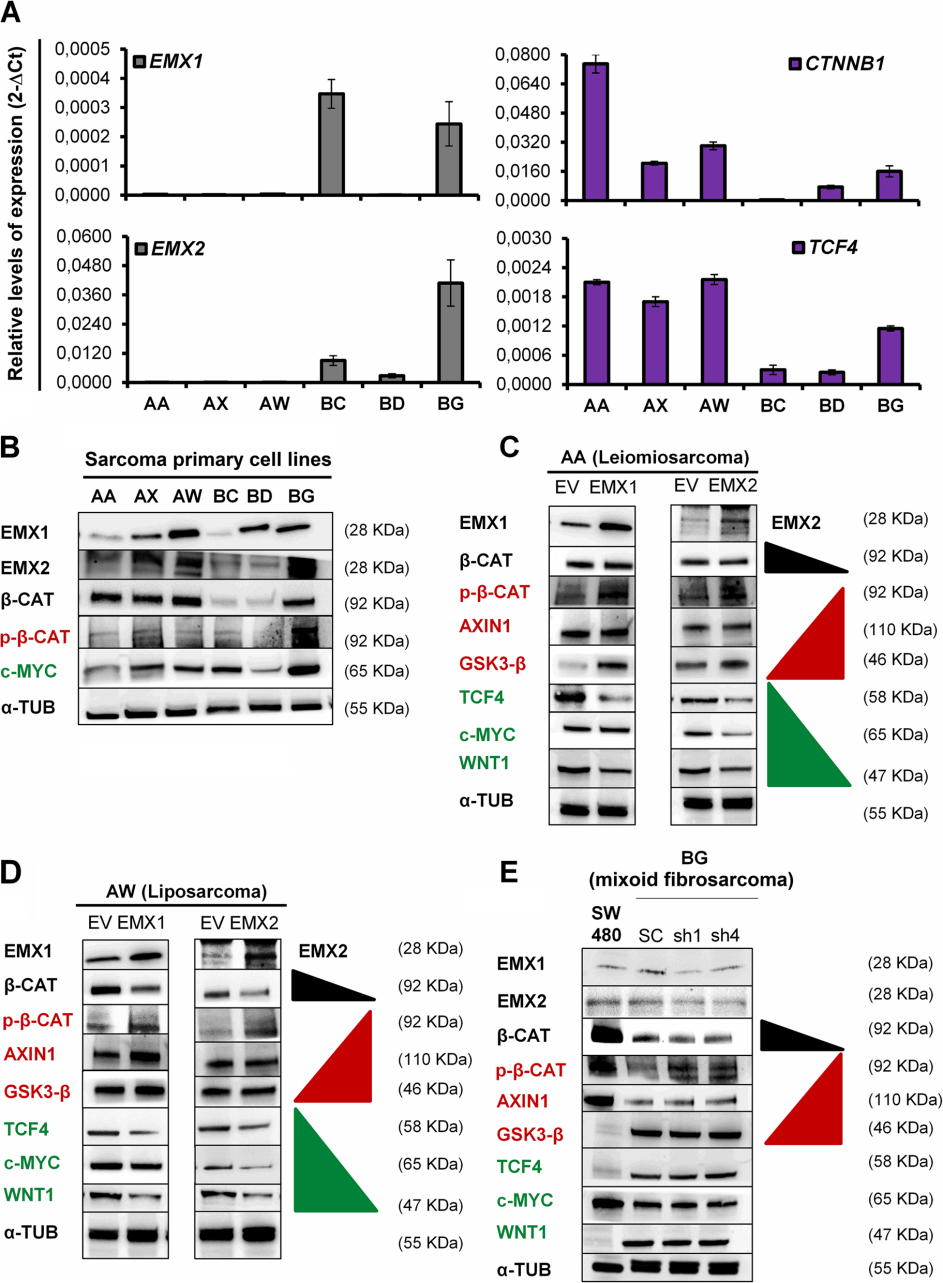
Transcriptional and protein characterization of the activation state of the canonical Wnt pathway in sarcoma cell lines depending on EMX expression. **A** Characterization of the expression levels of EMX1, EMX2, CTNNB1 and TCF4 in primary sarcoma lines. **B** Characterization of the levels of EMX1, EMX2, and the WNT pathway factors β-CAT (β-catenin), p-β-CAT (p-β-catenin S33/S37/T41) and c-MYC in the primary sarcoma lines. **C** and **D** Effect of EMX1 and EMX2 overexpression on the WNT pathway determined by analyzing the negative regulators p-β-CAT, AXIN1 and GSK3-β and the positive regulators β-CAT, TCF4, c-MYC and WNT1 in the AA (**C**) and AW (**D**) model cell lines. **E** Effect of reduced EMX1/EMX2 expression on BG cells. SW480 cells (colorectal cancer line) were used as a positive cellular control for inactivation of the Wnt pathway. A Western blot experiment representative of 3 experiments performed independently in triplicate is shown

The results suggest that in our model, there are functional relationships between the EMX1 and EMX2 genes and some of the pathways related to stem cell biology, especially the canonical Wnt/β-catenin pathway. From these previous analyses, we established 6 positive and negative effectors of the Wnt pathway as being directly related to the EMX1 and EMX2 genes at the transcriptional level. Therefore, we proceeded to characterize the activation state of the canonical Wnt pathway in primary sarcoma lines (Fig. 2A, B), including both EMX1 and EMX2 overexpression in AA and AW models (Fig. 2C, D) and EMX1/EMX2 silencing in a BG model (Fig. 2E). We measured β-catenin (β-CAT), TCF4, c-MYC, and WNT1 as positive effectors and the phosphorylated inactive form p-β-catenin S33/S37/T41 (p-β-CAT), AXIN1 and GSK3-β as negative effectors (Fig. 2).

The results indicated that there was activation of the canonical Wnt pathway in the primary AA and AW lines of parental sarcoma as these lines showed elevated protein expression of the dephosphorylated form of β-catenin, increased expression of the positive effectors CTNNB1 (β-catenin gene) and TCF4 and decreased expression of EMX genes (Fig. 2A, B). In contrast, there was inactivation of this pathway in the parental BG line, which was observed as increased protein expression of the phosphorylated and inactive forms of β-catenin, transcriptional reductions in CTNNB1 and TCF4 expression and increased expression of EMX1 and EMX2 (Fig. 2A, B). In the EMX1 and EMX2 overexpression models, clear inactivation of the pathway was observed (Fig. 2C, D). On the other hand, in the cell silencing model of both forms of EMX, there was a trend towards greater inactivation of the pathway, as indicated by an increase in the level of the phosphorylated and inactive form of β-catenin and a reduction in the level of the dephosphorylated form. In contrast, no differences in the positive effectors of the pathway, TCF4, c-MYC and WNT1, or the negative effectors AXIN1 and GSK3-β were observed (Fig. 2E).

**Fig. 3:**
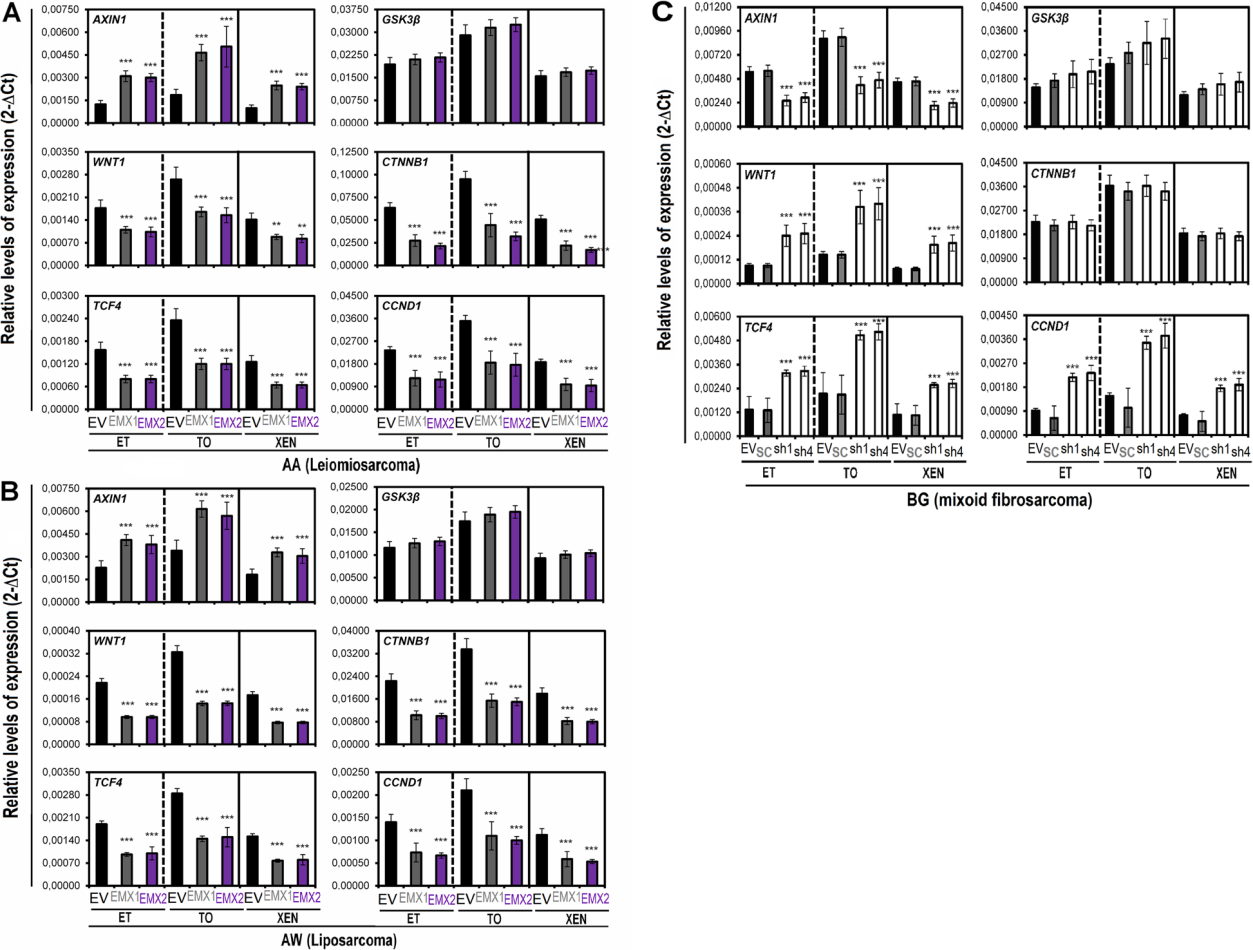
Effects of EMX1 and EMX2 levels on the expression of target genes in the Wnt signaling pathway. **A** and **B** Relative mRNA levels of the AXIN1, GSK3β, WNT1, CTNNB1, TCF4 and CCND1 genes determined by qRT-qPCR in AA (**A**) and AW (**B**) cell lines. **C** Effects of reducing EMX1 and EMX2 expression on the expression of the same genes related to the Wnt signaling pathway in the BG cell line. Graphs of the expression levels (2-ΔCt) for total extracts of each cell line (ET), tumorspheres (TO) and xenotransplants (XEN). The mean of a minimum of 3 independent experiments performed in triplicate ± standard deviation are shown. Statistical analysis was performed with Student’s t test (* *p* < 0.05; ** *p* < 0.01; *** *p* < 0.001)

These results measured at the protein level correlated with results measured at the messenger RNA level by qRT-PCR in the cellular models of overexpression and silencing (Fig. 3). We observed reductions in the levels of the positive effectors of the pathway and an increase in the level of the negative effector AXIN1 in the AA and AW cell lines, indicating that transcriptional inactivation of the canonical Wnt pathway occurs in the context of EMX1/EMX2 overexpression (Fig. 3A, B). On the other hand, increases in the levels of the positive effectors of the Wnt pathway and a reduction in that of the negative effector AXIN1 were observed in the BG cell line, indicating transcriptional activation of the canonical Wnt pathway in the EMX1/EMX2 silencing model (Fig. 3C).

These data confirm the negative functional relationship between EMX genes and WNT pathway activation.

### Effect of canonical Wnt pathway activation on EMX1/EMX2 overexpression

Overexpression of the EMX1/EMX2 genes inactivated the Wnt pathway and therefore could reduce tumorigenic properties and features related to the biology of mother cells. How does activation of the canonical Wnt pathway affect the tumor suppressor activity of the EMX1/EMX2 genes? To address this, a constitutive overexpression model of the Wnt pathway was created by ectopically overexpressing the β-catenin gene (CTNNB1) with mutations in the 4 phosphorylation sites. Therefore, this protein could not be phosphorylated and degraded, maintaining pathway activation in cells. Thus, we hypothesized that the expression of mutated and constitutively active β-catenin would recover the tumorigenic phenotype even when the EMX1/EMX2 genes were highly expressed.

**Fig. 4:**
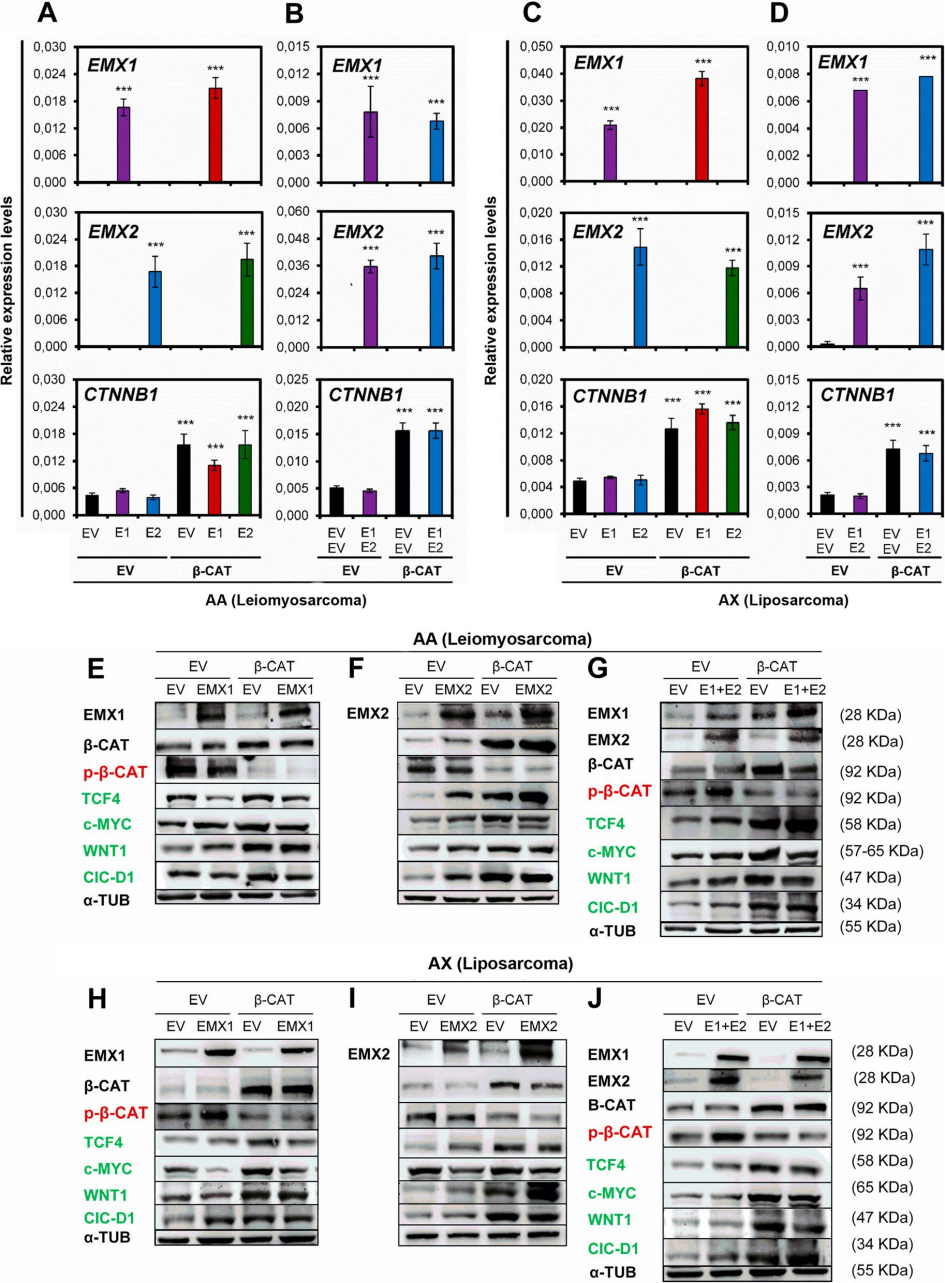
Effects of overexpressing EMX1 and EMX2 in combination with constitutive activation of the Wnt pathway. **A** and **C** Relative mRNA levels (2-ΔCt) of the EMX1 (E1), EMX2 (E2) and CTNNB1 (β-CAT) genes determined by qRT-qPCR in models of overexpression of EMX1 or EMX2 and CTNNB1 in AA (**A**) and AW (**C**) cell lines. **B** and **D** Joint overexpression of EMX1, EMX2 and CTNNB1 in AA (**B**) and AW (**D**) cell lines. The mean of a minimum of 3 independent experiments performed in triplicate ± standard deviation are shown. Statistical analysis was performed with Student's t test (* *p* < 0.05; ** *p* < 0.01; *** *p* < 0.001). **E** and **F** Characterization of the protein levels of EMX1, EMX2 and the WNT pathway factors β-CAT (β-catenin), p-β-CAT (p-β-catenin S33/S37/T41), TCF4, c-MYC, WNT1, and CIC-D1 (Cyclin D1) in simple models of EMX1 (**E**) and EMX2 (**F**) overexpression with constitutive activation of the Wnt pathway in the AA cell line. **G** Triple overexpression of EMX1, EMX2 and mutant CTNNB1 in AA cells. **H** and **I** Characterization of simple models of overexpression of EMX1 (**H**) and EMX2 (**I**) with CTNNB1 in the AW cell line. J, Triple overexpression of EMX1, EMX2 and mutant CTNNB1 in the AW cell line. A Western blot representative experiment of 3 experiments performed independently in triplicate is shown

To study this point, cell models of EMX1/EMX2 overexpression were generated with constitutively active mutated CTNNB1. Double (EMX1 or EMX2 + mutant CTXNB1) and triple (EMX1 + EMX2 + mutant CTNNB1) cellular models of overexpression were validated at the messenger RNA and protein levels (Fig. 4). We observed that the presence of individual EMX gene overexpression was not capable of inactivating the WNT pathway activation induced by the constitutively active mutant form of CTNNB1. The same behavior was observed in the triple overexpression model including EMX1 + EMX2 + mutant CTNNB1 (Fig. 4).

**Fig. 5:**
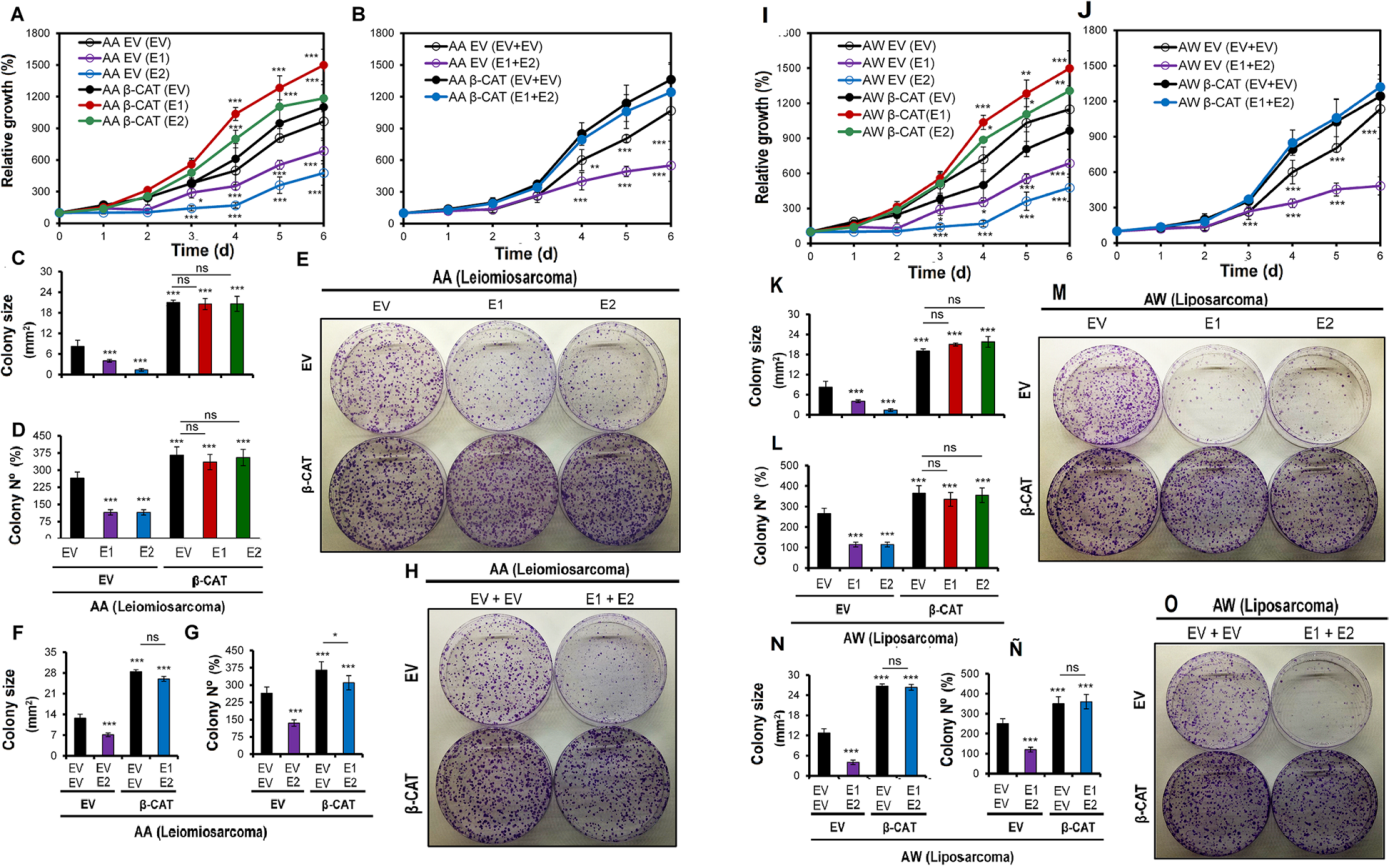
Effects of overexpression of EMX1 and EMX2 in combination with constitutive activation of the Wnt pathway. **A** and **B** Growth curves for AA cells with overexpression of EMX1 (E1) or EMX2 (E2) and CTNNB1 (β-CAT) (**A**) or joint overexpression of EMX1 + EMX2 + CTNNB1 (**B**). **C**-**H** Clone formation test performed with the same AA models. In **C** and **F** the size of the colonies is indicated. In **(D)** and **(G),** the graphs show the number of colonies. In **E** and **H** representative images of the clone formation assay plates stained with crystal violet on the last day of the experiment are shown. **I**-**O** same for the AW cell line. **I, J** Growth curves for AW cells with overexpression of EMX1 (E1) or EMX2 (E2) and CTNNB1 (β-CAT) (**I**) or joint overexpression of EMX1 + EMX2 + CTNNB1 (**J**). **K**–**O** Clone formation test performed with the same AW models. In **K** and **N** the size of the colonies is indicated. In **L** and **Ñ** the graphs show the number of colonies. In **M** and **O** representative images of the clone formation assay plates stained with crystal violet on the last day of the experiment are shown. The mean of a minimum of 3 independent experiments performed in triplicate ± standard deviation are shown. Statistical analysis was performed with Student's t test (* *p* < 0.05; ** *p* < 0.01; *** *p* < 0.001)

Additionally, canonical Wnt pathway genes were characterized using qRT-PCR (Fig. 5). A reduction in the relative expression level of the AXIN1 gene and increases in those of positive effectors (TCF4, WNT1 and CCND1) were observed in cells with double overexpression of EMX1 or EMX2 with mutant CTNNB1 (Fig. 5) and in cells with triple overexpression of EMX1 + EMX2 with mutant CTNNB1 (Fig. 5) in both the AA and AW cell lines. Likewise, the transcriptional expression levels of Wnt effectors were not altered in the presence of EMX1/EMX2 overexpression.

Therefore, since the EMX1/EMX2 genes are not capable of inactivating the Wnt pathway in the presence of constitutively active β-catenin, the downstream EMX pathway.

### Functional effect of EMX1/EMX2 overexpression in combination with constitutive activation of the Wnt pathway

Therefore, the results to this point suggest that β-catenin acts downstream of the EMX pathway. Taken together, the results suggest that EMX1/EMX2 regulate the Wnt pathway, since overexpression of EMX1/EMX2 inhibits the Wnt pathway, while constitutive activation of this pathway is independent of EMX1/EMX2 levels. However, it is still unknown whether the functional effect of EMX1/EMX2 is dependent on the Wnt pathway.

**Fig. 6:**
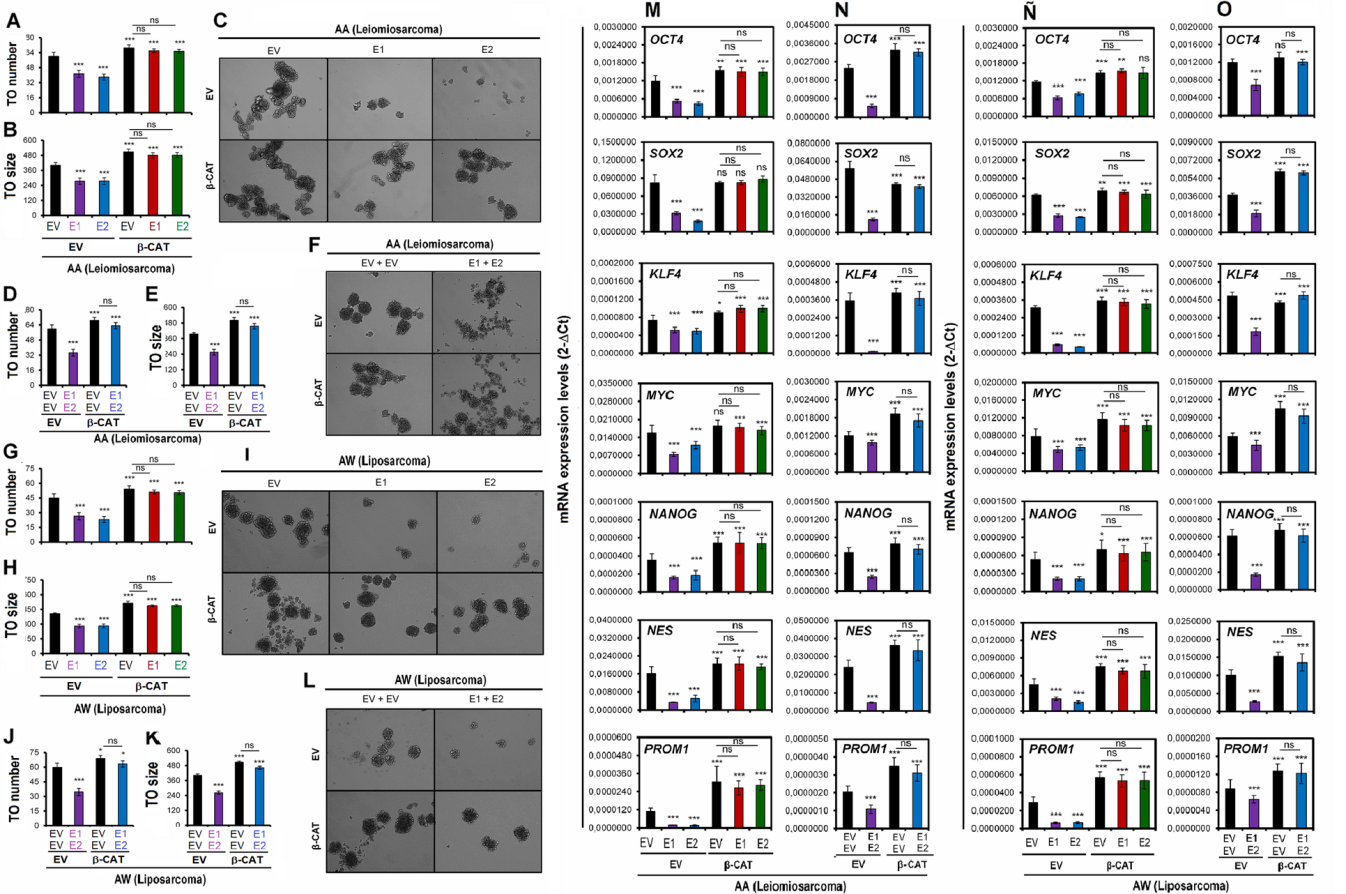
Functional effects of the overexpression of EMX1 and EMX2 in combination with constitutive activation of the Wnt pathway on tumorsphere (TO) formation. **A-L** Tumorsphere (TO) formation assay results for models of overexpression of EMX1 (E1) or EMX2 (E2) and CTNNB1 (β-CAT) in AA cells (**A**, **B** and **C**) and AW cells (**G**, **H** and **I**). Models of joint overexpression of EMX1, EMX2 and CTNNB1 in AA cells (**D**, **E** and **F**) and AW cells (**J**, **K** and **L**). In **A, D**, **G** and **H** the mean number of tumorspheres generated by AA cells (**A** and **D**) or AW cells (**G** and **J**) is represented. The mean sizes of the tumorspheres generated by AA cells (**B** and **E**) and AW cells (**H and K**). In **C**, **F**, **I** and **L** representative micrographs of the tumorsphere formation results for AA cells (**C** and **F**) and AW cells (**I** and **L**). The mean of a minimum of 3 independent experiments performed in triplicate ± standard deviation are shown. TO size is given as μM diameter. **M**–**O** Effects of EMX1 and/or EMX2 overexpression in combination with constitutive activation of the Wnt pathway on genes of the canonical Wnt pathway. **M**, **Ñ** Quantification of the relative mRNA levels of the AXIN1, GSK3β, TCF4, WNT1, and CCND1 determined by qRT-qPCR in AA cells (**M**) and AW cells (**Ñ**) overexpressing EMX1 or EMX2 and CTNNB1. **N** and **O** Quantification of the relative mRNA levels of the same genes in AA cells (**N**) and AW cells (**O**) jointly overexpressing EMX1, EMX2 and CTNNB1. Graphs of the expression levels (2-ΔCt) for total extracts of each cell line are shown. The mean of a minimum of 3 independent experiments performed in triplicate ± standard deviation are shown. Statistical analysis was performed with Student's t test (* *p* < 0.05; ** *p* < 0.01; *** *p* < 0.001)

Functional proliferation, clone formation, and tumor formation tests and measurement of the expression levels of genes related to stem cell biology were performed to evaluate the effect of the constitutively active Wnt pathway on the individual or combined overexpression of the EMX1/EMX2 genes (Fig. 6). We observed recovery of proliferation (Fig. 6A, B) and clone formation in number and size (Fig. 6E-O) in cells overexpressing EMX genes in the presence of the constitutively active mutated form of CTNNB1. Therefore, the tumor-suppressive effect of the EMX1/EMX2 genes measured in terms of the proliferative rate and clone formation is lost when mutant CTNNB1 is overexpressed.

In the same way, whether a behavioral reversal in the properties related to stem cells occurred was assessed (Fig. 6). A tumorsphere (TO) formation test was performed with the double and triple models of the AA and AW lines (Fig. 6A-L). In the context of overexpression of EMX1, EMX2 or EMX1 + EMX2 without the expression of mutant CTNNB1, reductions in the number and size of the tumorspheres formed were observed. However, the number and size of tumorspheres recovered with the addition of the expression of active CTNNB1 (Fig. 6A-L).

**Fig. 7:**
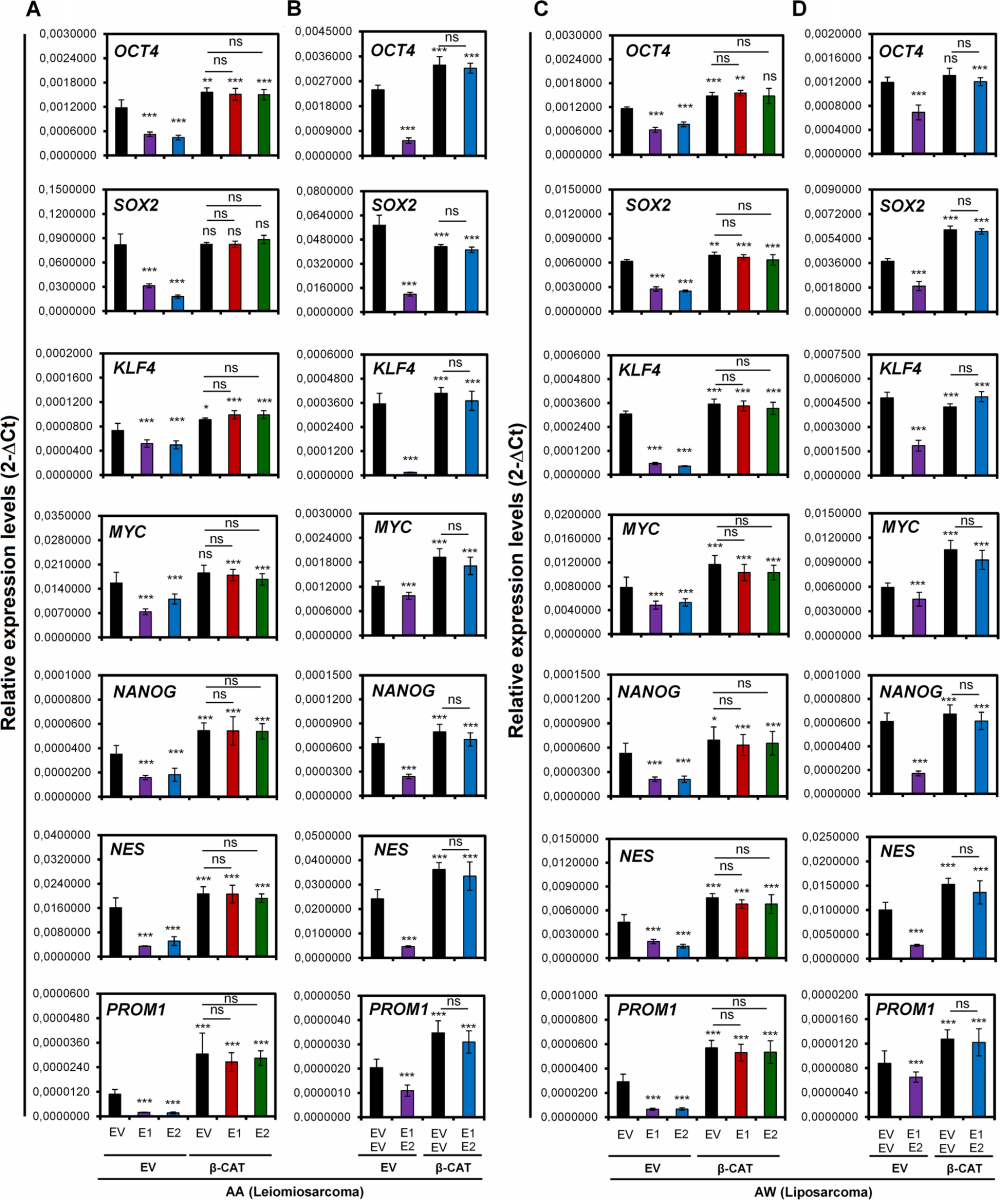
Effects of overexpression of EMX1 and/or EMX2 in combination with constitutive activation of the Wnt pathway on stem cell gene expression Quantification of the relative mRNA levels of the OCT4, SOX2, KLF4, MYC, NANOG, NES and PROM1 genes determined by qRT-qPCR in AA cells (**A**) and AW cells (**C**) overexpressing EMX1, EMX2 and CTNNB1**.** Models of joint overexpression of EMX1, EMX2 and CTNNB1 in AA cells (**B**) and AW cells (**D**). Graphs of the expression levels (2-ΔCt) for total extracts of each cell line. The mean of a minimum of 3 independent experiments performed in triplicate ± standard deviation are shown. Statistical analysis was performed with Student’s t test (* *p* < 0.05; ** *p* < 0.01; *** *p* < 0.001)

Additionally, the expression levels of stem cell genes (OCT4, SOX2, KLF4, MYC, NANOG, NES, and PROM1) were also restored in the presence of the active WNT pathway (Fig. 7A-D). These results show a clear recovery of stem cell-related properties in the presence of EMX1/EMX2 overexpression when the canonical Wnt pathway is constitutively activated. In this way, the Wnt pathway was established as one of the pathways related to the action of the transcription factors EMX1/EMX2 in sarcoma tumorigenesis.

## Discussion

Sarcomas are a large group of different malignant diseases and are very heterogeneous at both the intertumoral and intratumoral levels. Intratumoral diversity can be understood as part of clonal genetic evolution, which may include relationships between sarcoma cells. These relationships may be hierarchical and regulated by genetic and epigenetic factors, extracellular signals, particles, and intrinsic developmental programs derived from the sarcoma cells of origin. The alternative possibilities for sarcoma cells of origin, such as neural crest-derived stem cells, mesenchymal stem cells and mesenchymal committed precursor cells, are heterogeneous and may explain the substantial heterogeneity in sarcoma initiation and inter- and intratumoral sarcomas. Further genetic and epigenetic changes associated with sarcomagenesis profoundly affect the biology of sarcoma stem cells. Understanding the biology of sarcoma stem cell origin could improve sarcoma patient clinical care, leading to better patient stratification and, hopefully, the development of more effective therapeutic options.

Self-renewing sarcoma populations originate from neural or mesodermal precursors. EMX1 or EMX2, neural crest-regulating transcription factors, expression reduces tumorigenic properties, while reductions in their levels enhance these properties. Furthermore, these EMX genes decrease the expression of stem cell regulatory genes and the stem cell phenotype. Together, these results indicate that the EMX genes negatively regulate these tumor-remodeling populations or cancer stem cells, acting as tumor suppressors in sarcoma [4].

Additionally, the Wnt pathway is one of the mechanisms that explains the relationships between EMX and stem cell genes. EMX1/EMX2 reduce the activation of the Wnt pathway, properties of cancer stem cells, and expression of genes that regulate stemness. Furthermore, when the Wnt pathway is constitutively active, the stemness properties of cells are activated regardless of EMX1/EMX2 expression. These data suggest that the Wnt pathway is essential during the process of regulation of stem cells of neural crest origin and that its deregulation is strongly implicated in sarcomagenesis.

Despite the different molecular processes underlying sarcomagenesis, EMX genes are proposed to be transcription factors that can negatively contribute to tumors and are proposed as regulators of cell proliferation, differentiation, and migration, not only during brain development but also in the neoplastic transformation process of stem cells [4]. From analysis of 11 available datasets on the R2-Genomics platform, it was found that numerous signaling pathways regulating the properties of stem cells were related to EMX expression, including the canonical Wnt pathway. Correlation analysis of the 11 datasets revealed that 173 Wnt pathway genes were correlated with both EMX1 and EMX2. Therefore, the canonical Wnt pathway was proposed to be one of the permissive mechanisms that could explain the role of the new tumor suppressor EMX genes in sarcoma. In addition, this correlation is also supported by previous work that associated Emx2 and Wnt1 in the neural context [49] and found reduced Wnt pathway activity in the context of EMX2 overexpression in lung cancer, gastric cancer, glioblastoma, melanoma [20, 21, 50–52], and sarcomas such as Ewing's sarcoma, synovial sarcoma, leiomyosarcoma, osteosarcoma, and MPNST [37, 40, 53, 54].

In sarcoma cells, we found reductions in the protein levels of positive effectors of the Wnt pathway (β-catenin, TCF4, c-MYC and WNT1) and increases in those of negative effectors (GSK3-β and AXIN1) in cells overexpressing EMX proteins. Additionally, significant reductions in the levels of the positive effector genes of the Wnt pathway (CTNNB1, TCF4, MYC, WNT1 and CCDN1) and an increase in that of the negative effector AXIN1 were found at the transcriptional level under EMX overexpression. The opposite effects were also produced when the EMX1/EMX2 genes were silenced, indicating that the Wnt pathway is inactivated in the presence of EMX gene expression, as has been preliminarily demonstrated for EMX2 in other tumors, such as lung and gastric cancer [4, 15, 17]. When the expression of active mutant β-catenin was included in the context of expression of EMX1 or EMX2 individually or EMX1 + EMX2 jointly, the reductions in the levels of the positive effectors of Wnt disappeared, increasing the protein and transcriptional levels of the negative effectors of the Wnt pathway. In addition, it was found that coexpression of mutant CTNNB1 with EMX1 or EMX2 individually or both EMX genes (EMX1 + EMX2) enabled reversion to a more aggressive tumor phenotype due to increased cell proliferation and clonogenicity. Furthermore, increases in the properties of stem cells were measured by assessing the capacity for tumor formation and performing transcriptional analysis of the OSKM genes (OCT4, SOX2, KLF4 and MYC), NANOG, NES and PROM1. All of these results showed that the Wnt pathway was activated, restoring the tumorigenic phenotype and increasing the properties of stem cells, regardless of EMX expression. These results indicated a functional and regulatory relationship for the effect of EMX on the canonical Wnt pathway. This correlated with the distribution of these signaling pathways in the developing neural process [49]. Thus, regulating WNT1 could be the mechanism by which EMX genes can directly regulate the Wnt pathway. However, these genes may functionally interact with other WNT ligands or other pathway factors. This is supported by observations in lung [17] and gastric cancers [12, 15], where EMX2 and the Wnt pathway are also related. However, emphasis should be placed on future studies of EMX1/EMX2 as transcription factors to corroborate which gene sets can perform transcriptional regulate to thus propose a molecular model of the interactions of EMX1/EMX2 with the Wnt pathway, other stem cell genes and other related transcriptional programs.

In general terms, the expression of the EMX genes remains silenced or repressed in most bulk cells of tumors. The molecular mechanism underlying this suppression out is not known in detail, but hypermethylation of both promoters has been noted [12, 17, 55]. Hypermethylation of the EMX1 and EMX2 promoters has been demonstrated in primary sarcoma lines. This may be due to the functional correlation that the EMX1 and EMX2 genes exert their function in ontogenetic development but are silenced by hypermethylation of their promoters once their specific functions in the differentiation process have concluded. However, EMX expression might be maintained in only stem cells or progenitors of neural crest or mesodermal origin, maintaining quiescence by repressing the Wnt pathway. Upon EMX absence, Wnt becomes activated and *permissive* to further malignant transformation leading to development of sarcoma tumors. This hypothesis explains the increased rate of induced fibrosarcoma in EMX KO models [4] and the favorable prognosis of tumors with EMX expression [12, 13, 15–19, 56, 57]. Therefore, EMX overexpression could be considered a new therapeutic strategy for future gene therapy trials, as has been proposed in gastric cancer [12, 15].

## Conclusion

In summary, our work shows that EMX1 and EMX2 act as tumor suppressors by suppressing the activity of stem cell regulatory genes (*OSKM*, NANOG, and PROM1) and effectors of the canonical Wnt pathway (CTNNB1, TCF4, MYC, WNT1 and CCDN1) in sarcoma. Together with previous works, this study proposes that the expression of EMX1 and EMX2 is maintained in only cancer stem cells embedded in the tissue of origin of the sarcoma to maintain the quiescent state by repressing the Wnt pathway. Under these conditions, stem cells can become replicative or exit their quiescent state by repressing EMX transcription and subsequently activating the Wnt pathway. Upon genetic alteration and constitutive activation of the Wnt pathway, the expression levels of EMX genes become irrelevant, and these stem cells become cancer-initiating cells in sarcoma. These results also suggest the Wnt/b-catenin axis as a relevant therapeutic target in sarcoma.

## Supplementary Information

**Additional file 1: Supplementary Table 1**. List of plasmids used in our work. **Supplementary Table 2**. List of RT-PCR probes used in our work. **Supplementary Table 3**. List of antibodies used in our work.

## Data Availability

No datasets were generated during the current study. The datasets analyzed during the current study are publicly available in the different repositories, as indicated in M&M section.

## Abbreviations

Blast: Blasticidin
CSC: Cancer stem cells
DMEM: Dulbecco’s modified Eagle media
DMSO: Dimethylsulfóxide
EV: Empty vector
FACS: Fluorescence-activated cell sorting
FBS: Fetal bovine serum
FC: Calcium fosfate transfection method
F10: Ham’s F10 culture media.
GAPDH: Glyceraldehyde-3-phosphate dehydrogenase
G418: Geneticin
KEGG: Kyoto Encyclopedia of Genes and Genomes
MIFT: Microphthalmia associated transcription factor
EMX: Homeobox transcription factors homologous to the *Drosophila empty spiracles* gene
pRS: PRetroSuper vector
Puro: Puromicin
qPCR: Quantitative PCR
RPMI: Roswell Park Memorial Institute culture media
RT-PCR: Reverse transcription PCR
SC: Scrambled shDNA
SD: Standard deviation
shARN: Short hairpin RNA
TCGA: The Cancer Genome Atlas
WB: Western blot
WNT: Family of genes and proteins associated to genes wingless and int-1, wingless/integrated (Wnt) signaling pathway.
CTNNB1: Gene codifying for b-catenin protein
β-CAT: β-Catenin
p-β-CAT: Phosphorylated β-catenin at S33/S37/T41
c-MYC: Proto-oncogen related to oncogén retroviral aviar v-Myc
GSK3-β: Glycogen sintase 3-kinase b
TCF4: T-cell factor 4
