# Supplementary material for: Empty spiracles homeobox genes EMX1 and EMX2 regulate WNT pathway activation in sarcomagenesis
Source: J Exp Clin Cancer Res. 2021 Aug 7;40:247. doi: 10.1186/s13046-021-02048-9 (PMC8348834; doi:10.1186/s13046-021-02048-9)
Supplement: Supplementary file 1 — Additional file 1: Supplementary Table 1. List of plasmids used in our work. Supplementary Table 2. List of RT-PCR probes used in our work. Supplementary Table 3. List of antibodies used in our work. [file 13046_2021_2048_MOESM1_ESM.doc]

**SUPPLEMENTARY INFORMATION**

Supplementary Materias used in this work.

**Supplementary table 1: We used the following plasmid in our work**

| **Plasmid** | | **Insert Sequence** | | | **R. eukar.** | | **Origin** | |
| --- | --- | --- | --- | --- | --- | --- | --- | --- |
| pRS-SC sh | | - | | | Puro | | Origene  (TR313216) | |
| pRS-sh1-EMX1 | | GCTTCAATTTAAGCCACAGTGTCTCCGAG | | | Puro | | Origene  (TI363329) | |
| pRS-sh4-EMX1 | | GGCAGTCTCAGCCTCTCCGAGACGCAGGT | | | Puro | | Origene  (TI363332) | |
| pRS-sh1-EMX2 | | TCAAGCCATTTACCAGGCTTCGGAGGAAG | | | Puro | | Origene  (TI352857) | |
| pRS-sh4-EMX2 | | CGGTGGAGAATCGCCACCAAGCAGGCGAG | | | Puro | | Origene  (TI352860) | |
| pCMV6-EV | | - | | | G418 | | Origene  (PS100001) | |
| pCMV6-EMX1 | | *EMX1* ADNc | | | G418 | | Origene  (RC208006) | |
| pCMV6-EMX2 | | *EMX2* ADNc | | | G418 | | Origene  (RC222758) | |
| pLentiB-EV | | - | | | Blast | | Addgene  (#17451) | |
| pLentiB-EMX1 | | *EMX1* ADNc | | | Blast | | LAB | |
| pLentiB-EMX2 | | *EMX2* ADNc | | | Blast | | LAB | |
| pLentiP-EV | |  | | | Puro | | Addgene  (#39481) | |
| pLentiP-EMX1 | | *EMX1* ADNc | | | Puro | | LAB | |
| pLentiP-EMX2 | | *EMX2* ADNc | | | Puro | | LAB | |
| pLenti-GFP-EV | | *-* | | | - | | Addgene  (#19732) | |
| pcDNA3.1-EV |  | | Amp | G418 | | TI | | Invitrogen  (V79020) |
| pcDNA3.1-βcat(4Mut) | *CTNNB1 (*β-CATENINA) ADNc  4 mutatiions: S33/S37/T41/S45 | | Amp | G418 | | TI | | Addgene  (#29936) |

pRS: pRetroSuper; SC: random sequence sh-DNA (scrambled shDNA); EV: empty vector; A.: resistance; Amp: ampicillin; Kan: kanamycin; Pure: puromycin; G418: geneticin; Blast: blasticidin; T: transfection; LAB: generated in the laboratory.

**Supplementary table 2:** We used the following probes in our work

| **Human**  **Gene** | **Probe (human)** | **Mouse gene** | **Probe (mouse)** |
| --- | --- | --- | --- |
| *GAPDH* | Hs03929097_g1 | *Gadph* | Mm99999915_g1 |
| *EMX1* | Hs00417957_m1 | *Emx1* | Mm01182609_m1 |
| *EMX2* | Hs00244574_m1 | *Emx2* | Mm00550241_m1 |
| *Prom1* | Hs01009257_m1 |  |  |
| *NES* | Hs04187831_g1 | *Nes* | Mm00450205_m1 |
| *NANOG* | Hs04260366_g1 | *Nanog* | Mm02019550_s1 |
| *OCT4* | Hs00999632_g1 | *Oct4* | Mm03053917_g1 |
| *SOX2* | Hs01053049_s1 | *Sox2* | Mm03053810_s1 |
| *KLF4* | Hs00358836_m1 | *Klf4* | Mm00516104_m1 |
| *MYC** | Hs00153408_m1 | *c-Myc* | Mm00487804_m1 |
| *BMI1* | Hs00995536_m1 |  |  |

**Supplementary table 3. Membranes were incubated with the following primary antibodies:**

| **Antibody** | **Company** | | **Dilution** | | |
| --- | --- | --- | --- | --- | --- |
|  |  |  | **WB** | **IHQ** | |
|  |  |  |  | **DIL** | **DA** |
| **EMX1** (Rabbit polyclonal) | Abcam | ab136102 | 1:1000 | 1:100 | EDTA |
| **EMX2** (Mouse polyclonal) | Abcam | ab171818 | 1:1000 | - | - |
| **EMX2** (Rabbit polyclonal) | Invitrogen | PA5-34415 | - | 1:500 | TC |
| **α-tubulin** (Mouse monoclonal) | Sigma-Merck | T9026 | 1:10000 | - | - |
| **β-catenin** (Rabbit polyclonal) | CST | #8814 | 1:1000 | - | - |
| **p-β-catenin (S33/S37/T41)**  (Rabbit monoclonal) | CST | #9561 | 1:1000 | - | - |
| **c-MYC** (Rabbit monoclonal) | CST | #5605 | 1:1000 | - | - |
| **AXIN1** (Rabbit polyclonal) | CST | #2087 | 1:1000 | - | - |
| **TCF4** (Rabbit polyclonal) | CST | #2565 | 1:1000 | - | - |
| **GSK3-β** (Rabbit monoclonal) | CST | #9315 | 1:1000 | - | - |
| **WNT1** (Rabbit monoclonal) | Abcam | ab85060 | 1:1000 | - | - |
| **Cyclin-D1** (Rabbit monoclonal) | Abcam | ab16663 | 1:1000 | - | - |
| **KI67** (Rabbit monoclonal) | MAD | 000310-QD | - | 1:250 | TC |
| **Rabbit anti-mouse HRP** (polyclonal) | Abcam | ab97046 | 1:5000 | - | - |
| **Goat anti-rabbit** **HRP** (polyclonal) | Abcam | ab97051 | 1:5000 | - | - |
| **Goat anti-rabbit** **HRP** (polyclonal) | JAC | 111-035-003 | - | 1:400 | - |
| **Goat anti-rat** **HRP** (polyclonal) | JAC | 112-035-003 | - | 1:400 | - |
| **Rabbit anti-goat HRP** (polyclonal) | Abcam | ab97100 | - | 1:400 | - |

WB: *Western blot*; IHQ: Immunohistochemistry; DIL: Dilution; DA: Antigen unmasking; EDTA: Buffer EDTA; TC: citrate buffer; MAD: *Master Diagnostica*; JAC: *Jackson Immuno Research*, CST: *Cell Signaling Technologies;* SCB: *Santa Cruz Biotechnology*; Secondary antibodies in gray.
